# A real‐time freehand 3D ultrasound imaging method for scoliosis assessment

**DOI:** 10.1002/acm2.13709

**Published:** 2022-06-24

**Authors:** Weiwei Jiang, Xianting Chen, Chaohao Yu

**Affiliations:** ^1^ College of Computer Science and Technology Zhejiang University of Technology Hangzhou China

**Keywords:** 3D reconstruction, freehand 3D ultrasound, incremental imaging, real‐time imaging, scoliosis, spine ultrasound

## Abstract

Real‐time 3D ultrasound has gained popularity in many fields because it can provide interactive feedback to help acquire high‐quality images or to conduct timely diagnosis. However, no comprehensive study has been reported on such an imaging method for scoliosis evaluation due to the complexity of this application. Meanwhile, the use of radiation‐free assessment of scoliosis is becoming increasingly popular. This study developed a real‐time 3D ultrasound imaging method for scoliosis assessment based on an incremental imaging method. In vivo experiments involving 36 patients with scoliosis were performed to test the performance of the proposed method. This new imaging method achieved a mean incremental frame rate of 82.7 ± 11.0 frames/s. The high repeatability of the intra‐operator test (intraclass correlation coefficient [ICC] = 0.92) and inter‐operator test (ICC = 0.91) demonstrated that the new method was very reliable. The result of spinous process angles obtained by the new method was linearly correlated (*y* = 0.97*x*, *R* ^2^ = 0.88) with that obtained by conventional 3D reconstruction. These results suggested that the newly developed imaging method can provide real‐time ultrasound imaging for scoliosis evaluation while preserving the comparative image quality of the conventional 3D reconstruction method.

## 1 INTRODUCTION

Ultrasound has a large variety of clinical applications because it is portable, inexpensive, radiation free, and allows real‐time monitoring.^1^, ^2^ Compared with conventional B‐mode ultrasound, 3D ultrasound can allow direct visualization of the arbitrary plane of 3D volume and help obtain a more accurate view of the shape, size, and location of the organ and lesion.^1^, ^2^ The 3D ultrasound imaging method usually consists of three steps of scanning, reconstruction, and visualization, which are conducted separately.^3^ For the separation of the three steps, clinicians have to wait several minutes or even longer before obtaining any images, leading to time‐consuming and inefficient rendering. In addition, the separation limits 3D ultrasound to be used in a situation where immediate feedback is needed, such as intraoperative imaging.

To solve the above problems, real‐time 3D ultrasound imaging techniques have been developed and have gained popularity in many fields. Some publications have reported near real‐time imaging. Welch et al.^4^ used several scans to generate a data block and updated the volume with the block continuously. The slice rendering rate was reported to be 15 frames/s with a Silicon Graphics 320 workstation. The conventional reconstruction method pixel nearest neighbor (PNN) could be used for real‐time imaging, and the reconstruction and rendering rates were 23 and 12.5 frames/s, respectively.^5^ Edwards et al.^6^ achieved a speed of 12.5 frames using a replacement value pixel distribution method for reconstruction. Studies have also reported achieving real‐time imaging. Dai et al.^7^ explored the use of graphics processing units (GPUs) to complete incremental reconstruction and rendering at a speed of 26–58 frames/s. Chen and Huang^8^ designed a parallel computing method with the help of a GPU and achieved an imaging speed of up to 119 frames/s using Bezier interpolation.

With the development of hardware and reconstruction and rendering methods, real‐time 3D ultrasound imaging has become a promising technique in many clinical fields, including cardiology,^9^ surgical guidance,^10^, ^11^ musculoskeletal tissue,^12^ and vascular imaging.^13^ However, to the best of our knowledge, no comprehensive study has focused on real‐time 3D ultrasound imaging for scoliosis evaluation due to the complexity of the deformed spine, which involves complex geometry and multiple layers of soft tissue. There is a great demand for using 3D ultrasound imaging for radiation‐free assessment of scoliosis, particularly with real‐time feedback of image quality during imaging.

Scoliosis is a medical condition defined as a 3D spine deformity with lateral deviation and axial rotation of the vertebral column.^14^, ^15^ In general, scoliosis affects 1%–3% of the overall adolescent population and approximately 3% of adolescents in Hong Kong.^16^ In clinics, to monitor curve progression or evaluate treatment outcome, regular examination is needed, and standing X‐ray radiography is the gold standard.^17^, ^18^ However, frequent exposure to X‐ray could increase the risk of breast cancer^19^ and lung cancer.^20^ Compared with X‐ray, ultrasound is radiation free and suitable for frequent monitoring, and the use of ultrasound for scoliosis evaluation has received increasing attention over the past decade. Recently, freehand 3D ultrasound, allowing viewing body anatomy in 3D space, has been advanced by combining a conventional 1D array ultrasound probe with a position sensor,^21^, ^22^ and a number of such systems have been developed solely for scoliosis evaluation. One method is to manually mark the transverse processes in 2D ultrasound images with 3D positional data. The target images were acquired in real time through observations^23^ or selected from the acquired 2D image set.^24^ However, this method is relatively time‐consuming because each sonographic landmark has to be identified from dozens of B‐mode images. Another method is to form coronal spine images using different visualization methods, including the maximum intensity projection method^25^, ^26^ and the volume projection imaging method.^27^ In 2019, our group developed a fast 3D ultrasound projection imaging (FUPI) method for scoliosis assessment. In this method, reconstruction was performed, and the complete spine data were projected to obtain the coronal image. It was reported that the FUPI can greatly decrease the processing time (15.07 ± 0.03 s for FUPI vs. 130.31 ± 35.07 s for the conventional method).^28^ However, this method is still an off‐line imaging method and cannot be used in a situation where immediate feedback is needed. To solve this problem, a real‐time imaging method was developed in this study.

Compared with other applications, such as cardiology, real‐time 3D ultrasound imaging for the spine has two features. The first one is the relatively large input image size. The sizes in the reported publications were 552 × 274,^5^ 480 × 413,^7^ and 302 × 268.^8^ However, for spine imaging, the region of interest needs to be large enough to cover the whole spinous region with an input size of 640 × 480 in the transverse plane and scan over a much longer distance. The large size makes real‐time imaging difficult. The second feature is that most of the diagnostic information, such as scoliosis deformity curvature, is located in the coronal image. Therefore, this study aimed to provide coronal imaging with a real‐time display of images. The next sections are structured as follows. In Section 2, the proposed imaging method is described in detail, and we conduct in vivo experiments to demonstrate its performance. The experimental results are described in Section 3. The discussions and conclusions are presented in Sections 4 and 5, respectively.

## 2 METHODS

### 2.1 System description

**FIGURE 1:**
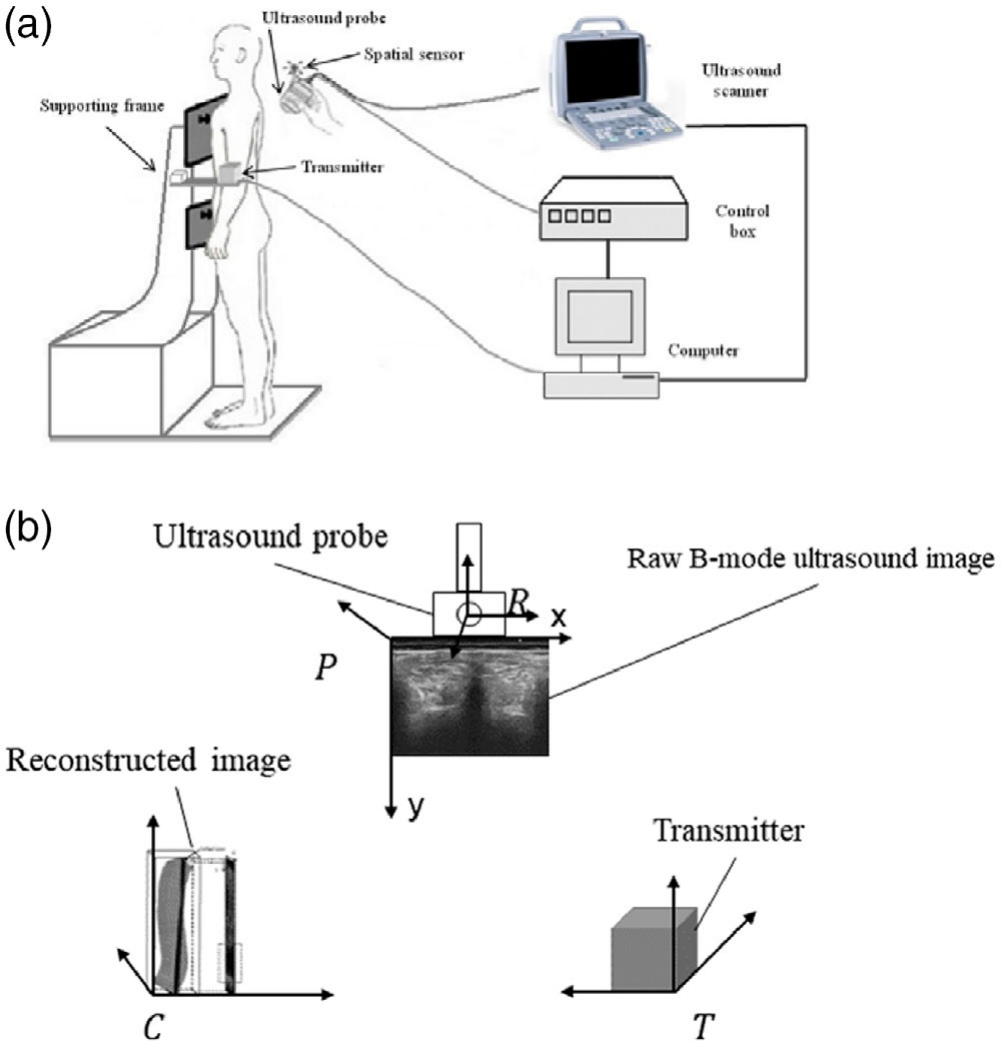
(a) The real‐time freehand 3D ultrasound system. (b) The four coordinate systems

A diagram of the real‐time ultrasound imaging system for scoliosis assessment is shown in Figure 1. The system consisted of an ultrasound scanner SIUI Apogee 1200V (SIUI Ltd., Guangdong, China) with a linear ultrasound probe, a compact electromagnetic spatial sensing device driveBAY (Ascension Technology, Burlington, VT, USA), a high‐performance computer with an Intel Core i5 3.35 GHz processor, and a custom‐designed supporting frame structure. The electromagnetic spatial sensing device comprised a control box, a transmitter, and a spatial sensor. The diameter of the cylindrical spatial sensor was 2.0 mm, and the length was 9.9 mm. It was mounted on the ultrasound probe to acquire 3D spatial data. The documented positional and angular accuracies of the spatial device were 0.5 mm and 0.1°, respectively, within the detection range of 46 cm. The supporting frame structure was designed to support patients steadily and to retain the spatial sensor within the designed working extent. A video capture card NI‐IMAQ PCI/PXI‐1411 (National Instruments Corporation, Austin, TX, USA) was installed on the computer and digitized B‐mode images with a maximum rate of 25 frames/s. A custom‐designed software programmed with C++ was run on the computer to acquire the B‐mode images and their corresponding spatial data for the subsequent rendering and visualization.

### 2.2 Real‐time imaging method

**FIGURE 2:**
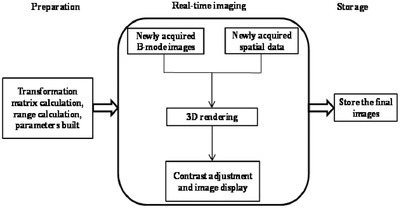
Flowchart of the real‐time imaging method

The flowchart of the proposed real‐time imaging method is shown in Figure 2, which consists of three steps of preparation, real‐time imaging, and storage.

As shown in Figure 1a, positional and orientational offsets exist between the spatial sensor and the ultrasound images obtained by the probe. Therefore, in the preparation stage, the first task was to determine the transformation matrix from the raw B‐mode ultrasound image to the coordinate of the reconstructed image. The matrix can be described as^29^:

(1) $${}^{c}T_{p}={}^{c}T_{t}•{}^{t}T_{r}•{}^{r}T_{p}$$

where *^c^T_p_* represents the transformation matrix from the coordinate system of subscript (*p*) to the coordinate system of superscript (*c*). As shown in Figure 1b, there are four coordinate systems, including the raw B‐mode ultrasound image (*p*), reconstructed image (*c*), transmitter (*t*), and receiver (*r*) of the spatial device. The transformation matrix *T* consists of three translation parameters (*t_x_*, *t_y_*, and *t_z_*) and three orientation parameters (*α*, *β*, and *γ*). The coordinate system of *c* is built by the user, so *^c^T_t_* can be known. The *^t^T_r_* can be read from the spatial sensor. To obtain the spatial relationship of *r* and *p* (matrix *^r^T_p_*), the spatial calibration needs to be performed.^22^, ^30^, ^31^ In this study, a cross‐wire phantom was used to conduct the calibration. Combined with *^c^T_t_* and *^t^T_r_*, *^c^T_p_* can be calculated.

During the preparation stage, the second task was to calculate the scanning range. Before scanning, the probe was placed at the bottom side (caudal to the fifth lumbar vertebra/L5) to record the lower boundary and then placed at the top side (cranial to the first thoracic vertebra/T1) to record the upper boundary. By combining an offset constant predefined by the operator, the cubic range of scanning could be calculated. In this method, the final coronal spine image was generated from a layer with a certain depth and thickness. The thickness was manually set and can be changed by the user before scanning. In this study, the value was set to 5.6 mm. The depth of the layer could be changed by the operator during scanning. In the preparation stage, all the arrays and variables, such as the index of raw images and voxel value array, were also built.

For the real‐time imaging stage, the whole procedure is shown in Figure 2. The newly acquired B‐mode images and the spatial data were sent to the computer. The spatial data were first transformed to obtain *^c^T_p_*. The images and *^c^T_p_* were transferred to the memory. A 3D rendering method was developed to generate coronal images based on the newly acquired data. Finally, after hole filling and contrast adjustment, the coronal image was displayed.

**FIGURE 3:**
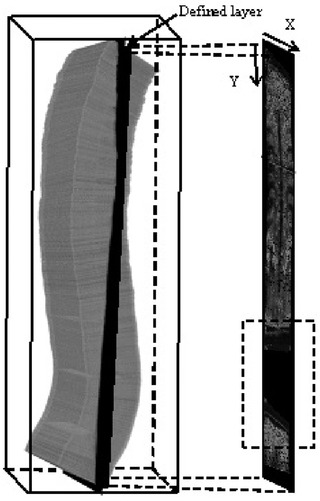
Illustration of the narrow‐band rendering method

In the stage of real‐time imaging, 3D rendering is the most important part. Conventional 3D rendering methods usually contain two steps: 3D volume reconstruction and image visualization. Unlike the conventional methods, the rendering method used in this study generated coronal images directly from the raw image data set instead of reconstructing the 3D volume. This rendering method, also called narrow‐band volume rendering, was first introduced by Gee et al.^32^ As shown in Figure 3, the rendering method first defined a layer with a certain thickness in the coronal plane. Meanwhile, a coronal image coordinate system with a regular pixel array was defined. The thickness of the defined layer could be set according to different applications. The layer depth was determined by the operator, and different coronal images at different depths could be obtained by changing this parameter. For the coronal image coordinate system, the density of the matrix in the *x*‐direction was determined by the ultrasound transducer, and that in the *y*‐direction was determined by the operator during rendering. Similar to the conventional reconstruction method, this new rendering method consisted of the bin filling stage and the hole filling stage. In the bin filling stage, a method similar to PNN interpolation was used. Each pixel of the raw data in the defined layer was transformed into the new coordinate system based on their spatial data and calibration matrix. The final value of the pixel on the newly generated image was calculated by averaging all pixels of the defined layer falling into its region. In the hole filling stage, each empty pixel was obtained by averaging the nonempty pixels in the nearest two‐by‐two neighborhood.

**FIGURE 4:**
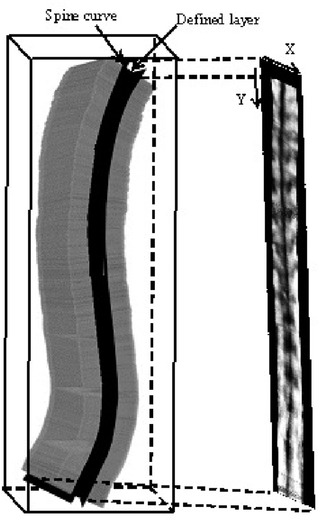
Illustration of the nonplanar rendering method

In Figure 3, the conventional planar reslicing method was used. However, some regions had no spinal bony features on the produced image because the natural curve of the spine (Figure 4) was not considered during the planar reslicing method. In this study, a nonplanar rendering method was introduced to follow the natural curve of the spine. A developable (unrollable) surface could be specified by the operator to follow the curved surface.^32^ The data set of Figure 4 showed that scanning followed the surface contour of the spine. Therefore, the developable surface could be determined based on the surface profile of the spine data set, which was also the physical surface of the ultrasound probe because the probe was in close contact with the skin during scanning. Therefore, a layer with a certain thickness and the same distance to the profile surface was cut from the raw data set (Figure 4).

**FIGURE 5:**
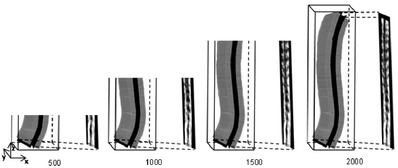
The diagram of the incremental imaging procedure when 500, 1000, 1500, and 2000 raw images are calculated

To perform real‐time imaging, an incremental imaging method was developed in this study. During spine scanning, the operator held the probe and steered it slowly to scan upward from the lower boundary (L5) to the upper boundary (T1). As shown in Figure 5, the acquired raw ultrasound images were roughly parallel to the plane of *xy* and along the direction of *z*, which could provide relatively regular raw images for incremental imaging. For the newly acquired B‐mode images, the new range *C*_new_ of these images was first calculated. The aforementioned 3D rendering process was performed based on the newly acquired images. For the coronal image, only the pixels in the range of *C*_new_ were updated. Finally, the contrast was adjusted based on the whole coronal image, and this image was displayed on the screen. Figure 5 demonstrates the incremental imaging procedure when 500, 1000, 1500, and 2000 raw images were acquired. On the basis of the incremental imaging method, only the updated raw data were computed in each rendering procedure, which could greatly save computation time.

When the upper boundary was reached, the real‐time imaging stopped, and the whole coronal image was automatically saved to the hard disk as a Bitmap image.

### 2.3 Subjects

Patients diagnosed with scoliosis were recruited from a tertiary scoliosis referral center in Hong Kong during March 2017. This study was approved by the local institutional review board. Informed consent was obtained from each subject prior to their enrollment in this study. The exclusion criteria were as follows: (1) body mass index (BMI) higher than 25.0 kg/m^2^; (2) with metallic implants; (3) patients who had received brace or surgical treatment; and (4) allergic to ultrasound gel during the scanning process. In total, 36 patients with scoliosis (mean age, 19.5 ± standard deviation 10.9 years; BMI, 19.4 ± 3.0 kg/m^2^) were recruited.

### 2.4 In vivo experiments

The proposed rendering method could provide coronal spine images while data acquisition was performed in real time. In vivo experiments based on patients with scoliosis were conducted to test the performance of the proposed method. During the test, the patient was requested to undress his or her upper garments and wear a dressing gown with an opening at the back for ease of scanning. All metallic wears and magnetic goods were removed to avoid influencing the electromagnetic spatial device. After the ultrasound gel was applied to the patient's back, the operator began scanning from the dorsal side of L5 upward along the spine until level T1 was reached by manually controlling the probe. The in vivo experiments included three parts of the imaging speed test, repeatability test, and comparison test between real‐time rendering and conventional off‐line reconstruction.

In this 3D ultrasound system, the sampling rate of the raw B‐mode image was approximately 25 frame/s, which limited the image generation rate of the proposed method and could not demonstrate the actual imaging capability of the system regarding real‐time processing. Therefore, in the imaging speed test, off‐line experiments simulating the real‐time imaging procedure were conducted to fully demonstrate the imaging speed limit. The patients were scanned following the procedure mentioned above. The size of the acquired B‐mode image was 480 × 640, and the number of frames in each raw data point was approximately 2500 for each patient. The acquired raw images together with their corresponding spatial data were recorded. The data were processed by the newly developed rendering method to generate coronal images of the spine. A timer was set to record the rendering time.

For the repeatability test, real‐time intra‐ and inter‐operator tests were performed. In the intra‐operator test, the patient was scanned by the first operator, and the coronal image was rendered and displayed in real time. After the whole scanning was completed, the complete spine coronal image was automatically recorded. The subject was asked to leave the standing position and relax for approximately 5 min. The same operator held the probe and conducted the second scan. In the inter‐operator test, the second operator scanned the subject, and the result was compared with the first data set of the intra‐operator test. In this test, three coronal images were obtained for each patient. Based on the coronal image, the spinal deformity could be measured using the spinous process angle (SPA) on the alignment of the spinous processes. As shown in Figure 4, the spinous processes had a dark profile in the middle of the image because the ultrasound could not penetrate through the bony structure. To mark the SPA, a pair of lines was manually drawn on the spine profile at the most tilted vertebrae region along the curve. If there were two curves, a third line was drawn at the lower end vertebra of the second curve. The software automatically calculated the SPA after the lines were drawn.

To analyze the performance of the proposed real‐time imaging method, a conventional 3D reconstruction method^27^ was used for comparison with the new imaging method. In the comparison test, all patients were scanned following the same scanning procedure. The reconstruction was conducted using the conventional method to generate the coronal image, which usually took 2–3 min. The SPAs based on these images were also measured. We compared the measurements obtained using the real‐time method and the conventional method separately.

### 2.5 Data analysis

For the imaging speed test, the mean rendering time was calculated. In the repeatability test, the intraclass correlation coefficient (ICC) values for the intra‐ and inter‐operator tests were obtained. The Currier criteria for evaluating ICC values were adopted: very reliable (0.80–1.0), moderately reliable (0.60–0.79), and questionably reliable (≤0.60).^33^ In the comparison test, the SPAs measured based on the real‐time imaging method and conventional reconstruction method were compared using a linear correlation. The Pearson correlation coefficient (*r*) was calculated to evaluate the correlation between the two methods. All statistical analyses were performed using statistical software (SPSS for Windows, version 24.0; SPSS, Chicago, IL, USA).

## 3 RESULTS

**FIGURE 6:**
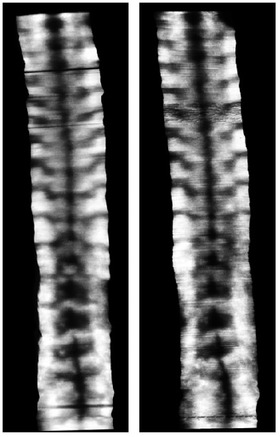
Typical comparison images from the conventional reconstruction and the new imaging methods for the same subject. Left: from the conventional 3D reconstruction method. Right: from the new real‐time imaging method

**FIGURE 7:**
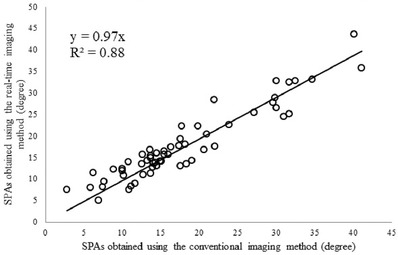
The correlation of spinous process angles (SPAs) measured using the images generated by the conventional imaging method and the developed real‐time method

In the rendering rate test, 36 data sets of patients (2711.3 ± 662.8 frames) were used, and the rendering time was 32.5 ± 4.8 s. The incremental frame rate of the real‐time reconstruction and visualization was 82.7 ± 11.0 frames/s. For the repeatability and comparison tests, 58 angles (SPA values: 18.3 ± 8.3°, range: 2.5°–44.5°) existed for the 36 patients. The repeatability in both intra‐operator test (ICC = 0.92) and inter‐operator test (ICC = 0.91) was found to be very reliable. In comparison with the conventional off‐line reconstruction method, the images formed by the new real‐time method were similar, as shown by the two typical images from the same subject in Figure 6. The two images showed similar spinous and shadow features, except the right image generated by the new method was sharper than the left image. Similar results were observed for images from all subjects. The correlation of SPAs measured on the images obtained from the two methods is shown in Figure 7, which demonstrated a very good correlation (*y* = 0.97*x*, *R* ^2^ = 0.88). Therefore, the new method had a similar ability to the conventional imaging method in illustrating spine deformities.

## 4 DISCUSSION

In this study, a real‐time 3D ultrasound imaging method for scoliosis evaluation was presented. An incremental imaging method based on narrow‐band rendering and nonplanar reslicing methods was used to realize real‐time imaging. The in vivo experiments on patients with scoliosis verified that this method could provide real‐time imaging and preserve the comparative performance in the produced projection images compared with the 3D reconstruction method.

In real‐time 3D ultrasound imaging, rendering speed is one of the most important performances that should be considered. The experiment showed that the speed of the developed imaging method was 82.7 ± 11.0 frames/s. With the typical acquisition rate of B‐mode images of 25–30 Hz, this method achieved real‐time ultrasound imaging. Many researchers have made extensive efforts to develop real‐time or near real‐time ultrasound methods. The reported imaging speed mainly ranged from 10 to 32 frames/s.^3,4^ In this study, the proposed method omits the step of 3D volume generation. This step takes most of the computing time because it needs the resampling process from the B‐scan to the voxel array in 3D space. In addition, the incremental imaging method, which only calculates the newly acquired raw images and updates the corresponding coronal image, can help save significant computation time. Therefore, the new method can achieve a higher incremental frame rate for real‐time imaging compared with conventional imaging methods.

In addition to speed, rendering image quality is another important factor for the proposed imaging method. The performance of in vivo scoliosis measurement is dependent on the quality of the rendering image. In this method, to obtain the complete image of the spine, a nonplanar reslicing method was used to follow the natural curve of the spine. The qualitative comparison between coronal images generated by the conventional method and the real‐time method showed that image quality was similar between the two images (Figure 6). Further quantitative comparison demonstrated a very good linear correlation (*y* = 0.97*x*, *R* ^2^ = 0.88) between SPAs obtained from images formed by the two methods. These results demonstrate that the image produced by the new method had a similar quality to the conventional method in illustrating the spine features. The rendering image quality is also highly dependent on the quality of the raw B‐mode images. However, it was found that the locating of spinal bony features for subjects with high BMI was a challenging task, which was ascribed to the poor image quality of ultrasound induced by the large attenuation of signals passing through the thick muscle and fat layers. Therefore, this study excluded people with BMI higher than 25.0 kg/m^2^ to obtain raw images with relatively high quality.

During the scanning of this study, the probe was moved slowly (approximately 1 cm/s). The scanning speed was displayed in real time in the software interface to help the operator control it. Therefore, sufficient raw B‐scan images were acquired, and a few holes needed to be filled for the reconstruction. In the proposed imaging method, PNN interpolation and bilinear interpolation were used in the bin filling and hole filling stages, respectively. The two stages adopting a plain process architecture can achieve a satisfying computation speed based on sufficient and regular raw data.

In some cases, such as surgery, fast scanning may be needed, which can cause sparse raw data. Therefore, further studies are needed to improve the rendering method to consider the interpolation accuracy and the computation time. Various reconstruction algorithms have been reported for 3D ultrasound reconstruction, including voxel nearest neighbor,^34^ pixel trilinear interpolation,^35^ and Bezier interpolation.^36^ Among them, kernel‐based algorithms, such as spherical kernel^21^ and 3D kernel,^7^ can provide more accurate reconstruction results. However, kernel‐based algorithms usually have a high cost of computing time because of the increasing computational complexity. To solve this problem, researchers have designed parallel computation using a GPU to decrease the computation time. Chen and Huang^8^ reported that the processing speed can be increased from 30 to 119 frames/s for Bezier interpolation and from 1.33 to 20 frames/s for squared distance weighted interpolation using a common GPU. Dai et al.^7^ also accelerated the reconstruction with the help of GPU to 90 frames/s. Therefore, in the future, the rendering method using the kernel‐based algorithm will be designed to improve the reconstruction accuracy. Meanwhile, the parallel computing technique based on a GPU will be developed to achieve real‐time imaging under fast scanning situations.

## 5 CONCLUSION

A real‐time 3D ultrasound imaging method for scoliosis evaluation is presented. The in vivo experiments demonstrated that this method could achieve real‐time imaging while preserving comparative imaging quality with conventional 3D reconstruction methods. With further improvements and large‐scale clinical tests, this real‐time imaging method is expected to facilitate scoliosis examination and surgery.

## CONFLICT OF INTEREST

The authors declare they have no conflicts of interest.

## AUTHOR CONTRIBUTIONS

*Conceptualization (lead), funding acquisition, supervision, and writing—original draft (lead)*: Weiwei Jiang. *Software (lead) and writing—original draft (supporting)*: Xianting Chen. *Software (supporting) and formal analysis (lead)*: Chaohao Yu.

## Data Availability

The data that support the findings of this study are available on request from the corresponding author. The data are not publicly available due to privacy or ethical restrictions.
